# The Biopolitics of Social Distancing

**DOI:** 10.1177/2056305120947661

**Published:** 2020-08-10

**Authors:** J.J. Sylvia

**Affiliations:** Fitchburg State University, USA

**Keywords:** COVID-19, biopolitics, social distancing, Michel Foucault, media genealogy

## Abstract

As COVID-19 spreads across the globe, new technologies are being leveraged to enforce social distancing requirements. I explore social distancing through the theoretical lens of Michel Foucault’s biopolitics, with an emphasis on recognizing unauthorized movement and controlling circulation. Although reporting and widely shared data visualizations about COVID-19 have made many people newly aware that their movements are being tracked and surveilled, governments are already implementing new measures such as geofencing and artificial intelligence (AI)–based facial recognition to facilitate the enforcement of social distancing. The tracking of COVID-19 spread and social distancing behaviors of the public has made more visible the practices of biopolitics but also generated new opportunities for even greater surveillance and control. The current moment offers an opportunity to shift public perceptions about data surveillance, technological control, and the racial disparities of biopower, much in the same way that public perceptions around social media shifted during and after the Arab Spring. How we collectively respond to these biopolitical processes will, in part, determine how such power relations are articulated in the future.

As COVID-19 spread across the globe in the beginning months of 2020, it occurred within the broader context of a decade-long shift in public perceptions related to social media and digital technologies. In 2010, social media platforms such as Twitter were widely celebrated as tools of liberation that could spread democracy around the globe, due in part to their use in the Arab Spring movement (Ghonim, 2013). Yet, as authoritarian governments began to understand how such tools were being used, they created new legal frameworks that both prevented those tools from being used by citizens in future revolutions and legalized surveillance practices that helped the regime maintain power (Connell & Vogler, 2017). This pandemic and the social distancing measures that have been utilized in order to reduce the spread of the virus have made the biopolitical practices of governments more visible than ever as they generate new opportunities for increased technological surveillance and control, especially as such measures are differentiated across particular segments of populations.

My past research has used the framework of media genealogy to explore how media affect our processes of subjectivation—how we are created as subjects (Sylvia IV, 2019). The COVID-19 pandemic is important because the policies, procedures, and management of social distancing regulations make personal some of the aspects of media genealogy that would otherwise remain theoretical to a general audience. In particular, Michel Foucault’s concept of biopolitics offers an important explanatory lens that can be used to analyze the COVID-19 pandemic. For Foucault, biopolitics is a way to describe the managing of populations through statistical, population-level regulations. Regimes of authority such as governments no longer have to rely only on disciplining specific, individual bodies. Instead, power must confront populations as political, scientific, and biological problems that must be managed en masse (Foucault, 2003, 2010) Examples might include the management of things like mortality rate or average life expectancy. This way of thinking about life also becomes part of each of our own processes of subjectivation—we are created as subjects within a society that applies biopolitical processes of population management. The COVID-19 global pandemic offers a chance to reflect on this aspect of our subjectivation because we can see clearly how ingrained it is in the way the virus’s impact is being discussed: the mortality rate is higher for those with preexisting conditions, children are less likely to die, seniors are at greater risk, and so on.

## Becoming Population

Beyond thinking about the virus in terms of populations writ large, the visualizations that have been created to show the effectiveness, or lack thereof, of social distancing measures raise questions about how such data are collected. In early April 2020, several news outlets reported on how students who were on spring break in Florida spread COVID-19 across the country as they returned home (O’Sullivan, 2020). In my own social media feeds, I immediately saw questions arise over exactly how such data were collected. In teaching about social media and big data, I have found that many people have a general understanding that digital advertisements are targeted, but lack any deeper understanding of or curiosity about exactly how those ads are targeted or what data are used in the process. Conversely, the detailed visualizations of maps that appear to indicate the movement of individual cell phones provoked curiosity and concern about data collection. Many were genuinely surprised to discover that third-party companies, such as X-Mode in this case, collect and make use of detailed location-based data for millions of cell phone users. This was the moment many first realized that their location data were *already* being collected and used for this, and likely other purposes, without their conscious knowledge of such practices. Understanding one’s own role in this data collection and visualization is a process of subjectivation wherein one consciously becomes part of the population that is being managed.

Governments around the globe have devised new ways of using data to enforce social distancing measures. The use of cell phone–derived location data has been the most common method of collection, with organizations such as the US-based Centers for Disease Control receiving a US$500 million grant via the Coronavirus Aid, Relief, and Economic Security (CARES) Act stimulus bill to support the development of better surveillance and data collection systems (Gershgorn, 2020; Holmes, 2020; Paine, 2020). These efforts have raised privacy concerns. Apple and Google collaborated to develop an application programming interface that facilitates contact tracing for third-party applications. They recommend that in order to protect privacy, developers use only local, de-centralized storage for any data that is collected. Ethicists have collaborated to develop a 12-factor system that could be used to evaluate the legal and ethical implications of such tracing systems (Morley et al., 2020). Collecting and using data about populations while protecting privacy is significant challenge.

The anonymization of these data is one of the factors that has garnered significant attention in the press. In April, *The Guardian* revealed a draft memo that raised concerns related to a COVID-19 app being developed by the United Kingdom’s National Health Service (NHS). This memo stated that device IDs could be used to de-anonymize data and allow individual app users to be identified, raising questions about whether this complies with current data privacy laws in the United Kingdom (Pegg & Lewis, 2020). In the age of big data, concerns associated with the possibility of de-anonymizing data are ever-present.

On the other end of the spectrum of privacy, Taiwan’s National Communication Commission created a cell phone–based geofencing system that explicitly targeted particular users who were quarantined (Hui, 2020). This system continually monitors cell phone locations, alerting authorities if the signal travels too far beyond the geofence to which the user is restricted. In addition, alerts are generated if the battery dies or the phone is turned off. While the ability to use location-based data is not itself new, its use during a pandemic offers an opportunity in which the public both becomes more aware of the use of these data and begins to normalize its use due to the extraordinary circumstances.

At the same time, relatively new uses of surveillance technology are being deployed. Both China and the United States have introduced drone technology in order to help monitor the spread of COVID-19. In China, drones are being used to carry large QR codes to cars at checkpoints, where they must scan the code and enter health information on a government website (Novak, 2020a). Cities in both China and the United States have used drones as portable loud speakers to enforce social distancing protocols (Sharma, 2020). In the United States, the town of Westport, Connecticut, deployed an even more advanced use of drone technology. These drones, powered by Draganfly software, can monitor an individual’s temperature, heart rate, and respiratory abnormalities, as well as the distance between individuals (Novak, 2020b). Connecticut’s Westport Police Department (2020) explained how the software functioned in a press release shared on Facebook: “The drone software uses biometric readings to understand population patterns and allows quicker reaction time to ongoing events or potential health threats.” This announcement drew immediate backlash, and within 6 hr, the department updated their press release to announce they would no longer be using the drones in this way.

Russia has also been public about its use of artificial intelligence (AI) facial recognition to combat COVID-19. The software is tied into the large network of cameras that are located on most buildings and are alleged to be able to instantly identify individuals violating their quarantine or social distancing measures (Rainsford, 2020). This use allows for population-level management that can be followed up with individual-based discipline measures. For example, one Russian citizen reported that police arrived at his home within 30 min of his taking out the trash, which violated his quarantine protocols.

Some of these technological solutions have been celebrated for their contributions to limiting the spread of COVID-19. Indeed, Foucault himself did not posit power, including biopolitical power, as inherently negative, but rather relational and thus always changing as circumstances change. From this perspective, we might choose to accept some of these measures of surveillance and control during a global pandemic even if we would not otherwise. However, the danger in such an acceptance is that authoritarian and democratic regimes alike may not repeal these uses after the pandemic has ended. It is therefore important to understand both the risks of such technology in terms of surveillance precedents and their broader impacts on our own processes of subjectivation.

## Racism, Biopower, and Responsibility

In the United States, news reports have revealed that COVID-19-related effects are occurring unevenly across racial lines. In New York City, for example, 80% of those who were ticketed for violating social distancing requirements are people of color (Morales & Joseph, 2020). Liz Mineo (2020) wrote about the double whammy the virus has had on Native American communities, who have suffered from relatively worse health impacts and financial disruptions. Serwer (2020) argues that once the racial disparity of these impacts became clear, the strategy and rhetoric for mitigating the virus also shifted: “The lives of disproportionately black and brown workers are being sacrificed to fuel the engine of a faltering economy, by a president who disdains them.” This can be seen in proposed regulations to protect business owners who reopen their facilities from lawsuits by workers who might get sick, with the meatpacking industry at the center of such disputes.

For Foucault, this racism is explicitly connected to biopower, which puts into practice biopolitics. He argues that the use of this biopower embeds racism into the fabric of the State:

Racism makes it possible to establish a relationship between my life and the death of the other that is not a military or warlike relationship of confrontation, but a biological-type relationship: “The more inferior species die out, the abnormal individuals are eliminated, the fewer degenerates there will be in the species as a whole, and the more I—as species rather than individual—can live the stronger I will be, the more vigorous I will be.” (Foucault, 2003, p. 255)

This racist reasoning can be seen quite clearly in the rhetoric criticizing stay-at-home restrictions and the closing of non-essential businesses, which are meant to better enable social distancing practices. To save the economy, many, including US President Donald Trump, have argued that the death of those who are most vulnerable is an acceptable sacrifice. Those most vulnerable to COVID-19 are seniors and Black, Indigenous, and People of Color (BIPOC). This line of reasoning culminated with Texas Lt. Governor Dan Patrick arguing that he would be willing to sacrifice his own life if it would contribute to saving the economy (Beckett, 2020). Through the lens of biopolitics, we can see that certain individuals are being asked to die so that others can continue to live, or, within the logic of biopower: “The very fact that you let more die will allow you to live more” (Foucault, 2003, p. 255). COVID-19 allows vulnerable individuals to be rhetorically re-appropriated as threats to the larger population.

Some US citizens have used these arguments to begin protesting stay-at-home orders, demanding that they be lifted despite the risks to those who are most vulnerable. These protests reveal biopolitical approaches adopted and supported not just by the State but also portions of its citizenry. It is biopolitics incorporated into processes of subjectivation. Dominique Memmi (2003) points to this shift as an emerging feature of biopolitics. She argues that in this formulation, citizens themselves are extended the ability and responsibility to make decisions about life and death through their own individual choices. This shifts the responsibility for the care of populations away from the government and onto the self-care practices of citizens themselves.

Yet, the decisions that must be made by individuals are done in the context of other policies and procedures already in place. For example, many workers in the United States are only able to afford access to health care through insurance policies offered by employers. Increased unemployment benefits are only available to workers who are not able to return to work because of COVID-19-related closures. However, some governors have reopened businesses in their states despite not seeing a significant decrease in the spread of the virus. The Wisconsin Supreme Court recently struck down Governor Tony Evers’ stay-at-home order, essentially opening the entire state up for business-as-usual. In these cases, the “choice” facing workers is to either return to a job that puts their life and the life of family members at risk, or to lose their jobs, their income or unemployment support, and their health insurance.

The COVID-19 pandemic lays bare the biopolitics of the current moment. On one hand, governments have increased measures of surveillance and control for population management. On the other hand, this occurs within the larger context of the racial disparities woven into biopower as individuals are increasingly asked to take responsibility for making life and death decisions themselves, especially in the United States. Although these trends have been present for decades, this moment makes these biopolitical practices visible and concrete to a much wider portion of the population. It offers an opportunity to shift public perceptions about data surveillance, technological control, and the racial disparities of biopower, much in the same way that public perceptions around social media shifted during and after the Arab Spring. How we collectively respond to these biopolitical processes will, in part, determine how such power relations are articulated in the future.
